# Toward precision music medicine in oncology care: reproducible, scalable and biomarker-driven interventions

**DOI:** 10.1016/j.eclinm.2026.104001

**Published:** 2026-06-11

**Authors:** Annelise Huynh, Kyle Noll, William J. Ray, Frederick F. Lang, Mei Rui

**Affiliations:** a Department of Neurosurgery, The University of Texas MD Anderson Cancer Center, Houston, TX, USA; b Department of Neuro-Oncology, The University of Texas MD Anderson Cancer Center, Houston, TX, USA; c Therapeutics Discovery Division, The University of Texas MD Anderson Cancer Center, Houston, TX, USA; d The Belfer Neurodegeneration Consortium, The University of Texas MD Anderson Cancer Center, Houston, TX, USA; e The Brain Tumor Center, The University of Texas MD Anderson Cancer Center, Houston, TX, USA; f Shepherd School of Music, Rice University, Houston, TX, USA; g Music-in-Medicine, The University of Texas MD Anderson Cancer Center, Houston, TX, USA

**Keywords:** Precision music medicine, Music-based interventions, Non-pharmacological cancer care, Neurophysiological mechanisms

## Abstract

A powerful modulator of the human stress response, music can substantially affect neurophysiological activities and target dysfunctional circuits implicated in various disorders including cancer. However, its clinical application has only recently garnered scientific attention. Cancer functions as both a disease and chronic stressor, triggering an inflammatory response and cascade of symptoms—including pain, fatigue, and cognitive dysfunction—prompting interest in supportive therapies that are non-invasive, low-cost, and scalable. Among these strategies, music-based interventions (MBIs) have shown promise. Whereas music therapy involves active guidance from a certified therapist, music medicine is a passive but immersive musical exposure without facilitation or active patient engagement. However, many studies conflate these modalities, limiting clarity and clinical translation. We focus distinctly on music medicine and discuss potential for application in oncology settings as a scalable and emerging empirically supported intervention capable of eliciting reproducible and measurable outcomes. We identify gaps in previous music medicine research, particularly its reliance on subjective endpoints, and recommend complementing these outcomes within a neurophysiological framework supported by objective assays incorporated into ongoing multidisciplinary, multimodal trials. We also propose integration of artificial intelligence and computational modelling to systematise outcome-specific music selection and validate music medicine's ability to modulate neurophysiological pathways involved in cancer-related symptoms.

## Graphical abstract

**Figure:**
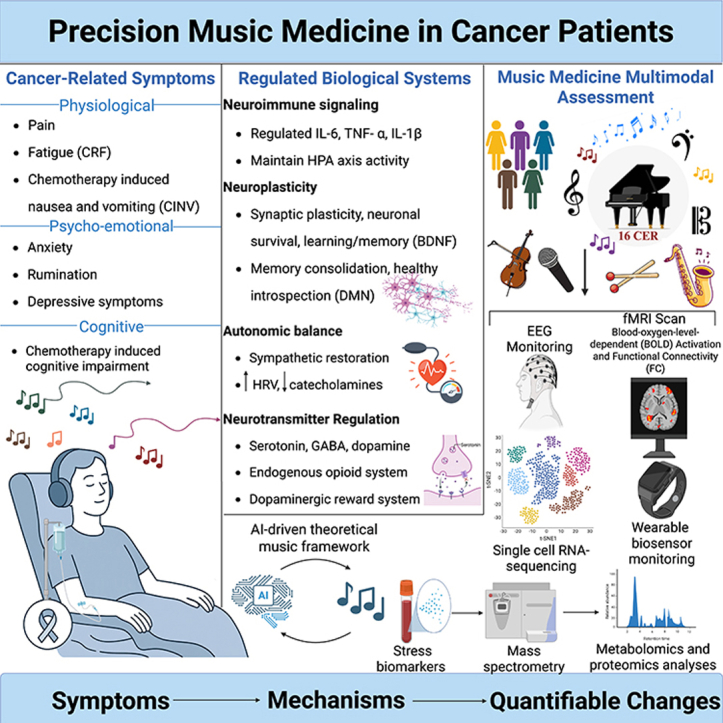

**Search strategy and selection criteria**

Data for this scoping narrative review were informed by the PRISMA Extension for Scoping Reviews and identified by searches of Cochrane Library, Web of Science, PubMed from 2000 to 2025 Because music medicine and music therapy are frequently conflated in literature, studies were classified according to intervention characteristics rather than nomenclature, consistent with the National Institute of Health-defined distinction between music medicine and music therapy and the operational definition of music medicine used herein. Eligibility criteria prioritised English-language, peer-reviewed randomised controlled trials, observational studies, and preclinical investigations evaluating music medicine interventions, defined as passive music listening without therapist involvement. Studies involving active music therapy, multimodal interventions in which the effects of music could not be isolated, or outcomes not relevant to cancer care were excluded. Included studies therefore used music as the primary intervention and assessed clinical, physiological, or symptom-related outcomes relevant to oncology.

Consistent with scoping review methodology, formal risk-of-bias or quality appraisal (e.g., JBI tools) was not performed. Additionally, as a scoping review the primary aim involved mapping existing evidence, characterising methodological variability, and identifying conceptual and translational gaps in the literature, rather than providing formal grading of evidence quality to inform reliability of pooled effect size estimates. Given the heterogeneity of study designs, interventions, and outcomes, a formal systematic review or meta-analysis was not feasible at present. Conclusions were therefore drawn through qualitative synthesis of recurring themes, areas of convergence, and methodological limitations across studies. The final body of literature spanned a wide range of journals, from high-impact general medical and neuroscience journals to speciality oncology, nursing, and allied health journals, reflecting the interdisciplinary nature of music medicine research.

## Introduction

Cancer remains the second leading cause of death in the USA, projected to affect more than 2 million people in 2025.^1^ Advances in detection and intervention have improved survival, yet chemotherapy, radiation, and surgery often inflict lasting side effects—pain, fatigue, anxiety, depression, and cognitive dysfunction—that persist beyond remission, impair quality of life, and impose substantial socioeconomic burdens.^2^^,^^3^ Thus, there is a pressing need to integrate safe, accessible, non-pharmacological strategies into standard cancer care. Music-based interventions (MBIs) have demonstrated promise in alleviating symptom burden across conditions with partially overlapping neurological and cognitive symptom profiles, including Alzheimer disease, Parkinson disease, fibromyalgia, and mood disorders.^4^, ^5^, ^6^ MBIs are increasingly adopted in oncology to target pain, anxiety, and fatigue due to their low cost, therapeutic impact, and adaptability.^7^

MBIs comprise two operationally distinct yet complementary modalities: music therapy and music medicine.^8^^,^^9^ Music therapy is a well-established clinical discipline involving active, relational techniques (e.g., improvisation, songwriting, emotional processing and interpretation) led by credentialed music therapists to address psychosocial and other functional goals. In contrast to music therapy, music medicine involves listening to pre-recorded or live music without therapist involvement. Both approaches have growing evidentiary support, including randomised controlled trials demonstrating the efficacy of structured protocols for symptom alleviation in neurologic, psychiatric, and other populations.^10^, ^11^, ^12^, ^13^ Recent work has also identified alterations in underlying neurophysiologic processes associated with the therapeutic effects of music therapy^14^^,^^15^ and music medicine.^16^^,^^17^ However, many studies conflate the two modalities, obscuring which intervention may be most appropriate for a specific clinical context and limiting understanding of mechanistic differences across treatment modalities. The following review surveys the literature specific to music medicine, which represents an MBI that can be easily disseminated in oncology settings, where time, resources, and patient engagement may be particularly limited. Music medicine demands no previous training and can be delivered by medical staff and caregivers, and scaled across diverse clinical and in–home settings.^18^ Potential inpatient applications include acute and critical care settings (e.g., intensive care units, pre-op rooms, radiotherapy suites), in addition to easy-to-implement outpatient interventions including take-home prescription music assignments. Music medicine's anxiolytic effects can extend to caregivers and appear beneficial regardless of education, musical background, or personal preferences^8^^,^^19^^,^^20^ with early indication of effects rivalling some pharmacologic approaches.^21^^,^^22^

However, critical gaps remain in standardising music medicine-based practices and elucidating the neurophysiological mechanism underlying their efficacy. Many music medicine studies continue to rely solely on subjective self-reported outcome measures and vaguely detailed methods that limit reproducibility. Furthermore, although some music medicine studies emphasise researcher-selected music to minimise emotional volatility,^23^ others employ patient-preferred music, which can complicate interpretability and generalisability of results. Notably, inappropriate music selections can evoke distressing memories or autonomic and haemodynamic instability (e.g., from intense vibrato or high frequency instrumentation). Additionally, overly activating music (death metal, hip-hop) could trigger arrythmias and haemodynamic instabilities in surgical settings.^24^ Unfortunately, few studies have assessed music medicine using multimodal, objective neurophysiological endpoints or examined its effects on inflammation, neuroendocrine signalling, or tumour biology. Together, these gaps in current music medicine studies reveal the lack of standardisation and mechanistic clarity that limit reproducibility.

This scoping narrative review addresses these gaps by presenting an expanded, empirically orientated definition of music medicine centred on objective neurophysiological and biomarker-based outcomes as the core underlying mechanisms of symptom reduction. Specifically, the review provides mechanistic clarity regarding emerging understanding of the biological and neurophysiological pathways engaged by music medicine, maps the existing evidence for music medicine applications in cancer care, and identifies methodological and conceptual gaps. The proposed neurophysiological pathways discussed in this review should be interpreted as preliminary mechanistic frameworks intended to guide future research, rather than as conclusively established causal mechanisms. Nonetheless, synthesis of this developing literature can be considered hypothesis-generating and understanding of current methodological limitations enabling of future more definitive work. Critically, rather than framing music as a solely experiential or emotional intervention, the perspective forwarded in this review posits music medicine as a powerful tool capable of both alleviating symptom burden and potentially modulating neurophysiological pathways implicated in cancer progression, while situating music medicine within the broader landscape of MBIs.

## Effects of MBI on cancer-related symptoms

### Physiological effects

Cancer and its treatments frequently induce a cascade of physical symptoms, such as pain, fatigue, and chemotherapy-induced nausea and vomiting (CINV) that impair quality of life and complicate treatment adherence.^25^ As pharmacologic strategies alone are insufficient to manage these symptoms, music medicine-based interventions have gained support for their capacity to attenuate symptom burden via various physiological and neurochemical pathways.^26^ Specifically, these interventions interact with complex neural circuits that regulate nociception, fatigue, and chemotherapy-induced nausea and vomiting (Table 1).

**Table 1:** Summary of music medicine interventions targeting pain in cancer care.

	Music intervention	Music selection	Control group	Main findings	Music therapist present?
Arruda et al.^27^	Passive listening; 3 days via MP3	Instrumental music	Standard of care; patients wore headphones	Music significantly reduced pain (p < 0.001) control group showed no significant change	No
Huang et al.^28^	Passive listening to “sedative” music for 30 min via headphones	Folk songs, Buddhist hymns, harp/piano; 60–80 bpm; no lyrics;	Resting in bed for 30 min	42% of music group achieved ≥50% pain relief vs 8% of controls	No
Zengin et al.^29^	Passive listening to researcher-selected played during port catheter placement procedure	Turkish classical music (Acemişiran); instrumental, slow	Standard care	Significantly lower mean VAS pain score in music group (3.14) vs control group (3.86); music reduced intraoperative pain during invasive procedure	No
Li et al.^30^	Passive listening to a 30 min sessions twice daily via MP3 and headphones, from first postop day through two chemo admissions	Library of 202 pieces (Chinese folk, Western, AAMT-recommended, relaxation pieces)	Standard care	Music significantly reduced PRI-total, VAS, and PPI scores at all three post-tests; effects persisted across hospital stay and chemotherapy admissions	No
Bilgiç et al.^31^	Passive listening via MP3 player and headphones; at least one time per day, three times per week, during chemo and 1 week post	30-min generic “relaxation” track (wave sounds + harp/violin)	Standard care	Music group reported significantly less increase in pain post-chemo (mean change = 0.229) vs control group (mean change = 2.000).	No

AAMT, American Association for Music Therapy; PPI, Present Pain Intensity; PRI, Pain Rating Index; VAS, Visual Analog Scale.

#### Effects of MBI on pain

Studies assessing music medicine for acute or procedural pain remain methodologically diverse, varying across pain models, intervention designs, music selection, and outcome measurement. These methodological differences complicate cross-study comparison and limit firm conclusions regarding efficacy or best practices, underscoring the need for more rigorous and standardised music medicine protocols. Although findings across the broader music-and-pain literature are not uniformly positive, several studies report reductions in mean pain scores relative to control conditions, suggesting that music medicine may hold promise as a low-risk adjunct for pain management in selected clinical contexts. Importantly, none of the studies required active facilitation by a music therapist but instead deployed researcher-selected music with calming acoustic properties. These findings may support music medicine's feasibility for patients with high symptom burden, particularly when fatigue or distress precludes active engagement. However, most investigations rely exclusively on subjective pain ratings, leaving unresolved whether the reductions reflect physiological modulation of nociceptive physiology or solely perceived discomfort. Future study designs would benefit from incorporating objective physiological assessments, such as analgesic consumption or biomarker shifts, in parallel with validated self-report pain measures to examine the extent to which music can reliably modulate the neurophysiologic signature and experiential manifestation of cancer-related pain.^32^

#### Effects of MBI on cancer-related fatigue

Cancer-related fatigue (CRF) significantly impairs cancer patients' physical function, emotional health, and quality of life.^33^ Preliminary trials show that passive listening can produce modest subject improvement in fatigue, and recent neurophysiological data from non-cancer populations demonstrate that instrumental music can reverse fatigue-associated alterations in brain activity by increasing individual alpha peak frequency (iAPF)—a marker of improved cognitive efficiency—and simultaneously reducing theta and alpha power across frontal, parietal, and occipital regions, a pattern consistently associated with fatigue mitigation.^34^ These patterns align with cognitive and attentional restoration, suggesting a plausible neural mechanism by which music medicine may alleviate CRF. Future work should investigate music medicine's neurophysiological mechanisms in CRF and identify musical compositions that optimally modulate EEG patterns linked to fatigue and pain. music medicine's potential to alleviate CRF remains underexplored, with existing studies typically using generic “relaxing” genres rather than standardised acoustic parameters linked to the greatest therapeutic effects, limiting reproducibility and precision for future research.

#### Effects of MBI on CINV

CINV remain among the most burdensome treatment side effects that undermine treatment adherence and quality of life of cancer patients.^35^ Early evidence supports music medicine as a promising, mitigatory intervention for CINV, though mixed results have been reported due to variability in baseline symptom severity, emetogenicity of chemotherapy regimens, and dosing of music exposure. In particular, Khuntee et al. (2022)^36^ reported no clear benefit of music medicine for postembolisation syndrome after receiving trans arterial chemoembolisation, largely because patients received a low-emetogenic agent (Mitomycin C) and exhibited minimal baseline nausea, leaving little room for measurable improvement.^37^ By contrast, trials with repeated exposure to music and higher baseline symptom burden demonstrated more favourable outcomes.^38^^,^^39^ These reductions in CINV can be delivered by providers and/or caregivers without requiring board-certified music therapists; a 20-min session of relaxing pre-recorded music (with optional at-home listening) significantly reduced anticipatory nausea and vomiting across 12 oncology clinics,^40^ with effects comparable to a more intensive mindfulness training protocol that requires greater time investment and patient engagement.

Together, these findings support a role for music as an adjunctive, low-burden, and scalable intervention for pain, CRF, and CINV, particularly relevant for patients with known anticipatory triggers or poor pharmacologic response. Despite promise, the noted methodological challenges the need for more studies to elucidate music's therapeutic efficacy for these common cancer-related symptoms.

### Psycho-emotional symptoms

Cancer patients frequently experience debilitating psychological stress, including anxiety, depressive symptoms, and other mood and personality changes, which impair quality of life and can interfere with treatment adherence.^38^ These symptoms may be present early in the disease course and may be particularly exacerbated by common procedures and treatments, including chemotherapy, bone marrow biopsies, and stem cell transplants, with up to 75% of patients reporting co-occurring anxiety and depression.^41^ Table 2 summarises evidence across music medicine interventions targeting these psycho-emotional outcomes.

**Table 2:** Summary of music medicine interventions targeting depressive symptoms and anxiety in cancer care.

	Music intervention method	Music selection	Control group	Main findings	Music therapist present?
**Depressive symptoms**					
Fernando et al.^42^	28-min music session via MP3 and earphones, eyes blindfolded	Instrumental classical music, composed by Sri Lankan composers	Standard care	Music significantly improved mood (p = 0.007), lasting up to 12 h; smaller pupils aligned with mood boost.	No
Jasemi et al.^41^	20-min daily music session for 3 days via Walkman and headphones.	Light, relaxing music, including sounds like sea, rain, and water	Standard care	Significant reduction in depression levels in the intervention group over 3 days (p < 0.001). Control group scores remained unchanged.	No
Lima et al.^43^	30-min pre-chemo music via MP3 and headphones, with brief relaxation guidance	Relaxing tracks, including classical music e.g., Yiruma, Franz Liszt	Standard care	Depression decreased in the intervention group (P = 0.04); All GM participants reported improved mood.	No
**Anxiety**					
Yang et al.^12^	Passive listening for 10 consecutive days	“Relaxing” piano and violin music	Standard care	Significant reductions in anxiety across BAI-C (p < 0.0001), BSRS-5 (p = 0.0024), and DT scales (p < 0.0001) in the music group compared to control	No
Jasemi et al.^41^	Music listening via headphones ≥20 min/day over 3 days	Light music (e.g., sea, rain, water sounds)	Standard care	Significant daily reduction in anxiety scores (p < 0.001) in intervention group across 3 days; no significant change in control group	No
Nguyen et al.^36^	20 min progressive music relaxation + 20 min music daily for 3 weeks via audio files.	Curated list (folk, religious, bolero) 60–80 bpm, instrumental, consistent rhythms, soft melodies	Standard care	Greater reductions in anxiety in intervention group at 3 and 6 weeks; participants reported feeling relaxed and calm, though results were not statistically significant	No
Chen et al.^44^	Single 15-min passive music listening session before radiation therapy.	Slow-paced, soft, melodic music at 60–80 bpm	Standard care	Significant reduction in both State and Trait anxiety in the music group vs control (p < 0.001)	No
Firmeza et al.^45^	Passive listening (30 min, via MP3 and headphones); volume self-controlled	‘Spring’ from Vivaldi's The Four Seasons; chosen for 60–70 bpm, pleasant tonalities	Standard care	Significant reduction in anxiety in the music group vs control; 100% of music group improved vs 75% of controls (p < 0.001)	No
Li et al.^30^	MP3 player and headphones; 30 min twice daily.	Media library (202 tracks, Chinese and world music)	Standard care	Significant reduction in state anxiety scores in music group compared to control at all post-tests; effect increased over time (p < 0.0001)	No
Mou et al.^46^	Wireless headphones during and after PICC placement; 30-min sessions	Classical, light, or folk music libraries; tempo ≤80 bpm, volume 45–60 dB, slow rhythm, low tone, soothing melody	Standard care	Significant reduction in anxiety in the music group at all time points; control group's anxiety increased during catheterisation (p < 0.001)	No
Karadag et al.^47^	MP3 player during daily radiation therapy for 5 weeks (20–40 min/session)	Bach's Trio Sonatas	Standard care	Significant reduction in HAD-A anxiety scores in music group; scores decreased while control group scores increased (p < 0.001)	No

BAI-C, Beck Anxiety Inventory, Chinese version; BSRS-5, 5-item Brief Symptom Rating Scale; DT, Distress Thermometer; GM, Group Music; HAD-A, Hospital Anxiety and Depression Scale–Anxiety subscale; PICC, Peripherally Inserted Central Catheter.

#### Effects of MBI on depressive symptoms

Fewer studies address the efficacy of music medicine interventions in attenuating depressive symptoms (Table 2) compared with active, therapist-delivered music therapy studies investigating active music engagement and the therapeutic relationship in reducing depressive symptoms.^48^, ^49^, ^50^, ^51^ Nonetheless, brief 20–30 min listening sessions before chemotherapy have reduced depressive symptoms more effectively than verbal relaxation controls,^43^ and similar improvements have been observed in HADS scores across three-day listening protocols using slow-tempo music though the short intervention left long-term relief uncertain.^41^ Non-oncology trials demonstrate that slow-tempo (70–80 bpm) music led to greater reductions in depression than quiet rest, and similar results have been observed across other non-cancer studies.^51^^,^^52^ However, most studies use loosely defined “relaxing” genres or patient-preferred music,^53^ though as noted above this non-standardised approach introduces bias and limits researchers’ abilities to attribute improvements to specific acoustic and structural properties of music. In addition to greater methodological harmonisation, the field would benefit from longer-term longitudinal studies to expand on the observed short-term results.^41^^,^^42^

#### Effects of MBI on anxiety

Music-based interventions have evidenced anxiolytic effects across oncology settings, with music medicine studies demonstrating that passive music listening significantly reduces anxiety in patients during medical procedures^12^ (Table 2). Anxiety is often driven by anticipatory fears, treatment side effects, waiting periods, unfamiliar settings and equipment, and isolation, and is implicated in treatment interruptions in up to 25% of patients.^54^

Importantly, none of these studies required a long-term therapeutic relationship with a provider, supporting broad scalability and disseminability of the interventions. Anxiety poses a direct challenge in diagnostic imaging, and this distress is magnified in cancer patients, for whom imaging is not only frequent but linked to emotionally laden stages of the disease course, such as diagnosis, staging, and treatment monitoring.^55^^,^^56^ Even in the absence of previous psychological diagnoses, up to 30% of patients undergoing MRI report acute anxiety.^57^ As such, heightened arousal blood pressure, respiratory rate, and muscular tension frequently lead to excessive motion, poor image quality, and prolonged patient exposure to radiation (Table 3).^58^^,^^56^ In PET-CT, stress-induced physiological changes can cause falsely elevated FDG uptake in muscle tissue, while functional MRI scans are susceptible to signal instability from head motion.^58^ Unlike informational videos, which may inadvertently heighten anxiety.^59^ Music medicine can reduce both anticipatory anxiety and sympathetic arousal, and its passive, immersive quality makes it ideal to redirect patient focus away from ruminative thought patterns and limit movement.^8^ Notably, Walworth (2010)^60^ reported that patients receiving live music intervention during MRI scans demonstrated significantly improved scan experiences, with fewer movement-related scan repetitions required, reduced break requests, and shorter scan times compared with the control group, evincing music's capacity to modulate somatic and affective responses that may interfere with the clinical integrity and efficiency of imaging procedures across diverse clinical settings.

**Table 3:** Summary of music medicine interventions for movement during imaging procedures.

	Music intervention	Music selection	Major findings	Imaging quality
Lee et al.^61^	30-min music listening session played via CD players and individual headphones before imaging procedures	60–80 bpm, instrumental, 50–60 dB	State Anxiety (STAI-S), heart rate decreased (p < 0.001). Music was beneficial during anticipatory anxiety phase before PET.	Potential reduction in false positives due to decreased sympathetic activity
Martinez-Lorca et al.^58^	50–60 min of music listening D via headphones and MP3 players during PET-CT uptake phase	Dan Gibson's nature-classical mix (slow tempo, natural sounds) delivered	STAI-State, subjective anxiety, systolic BP, diastolic BP, heart rate all significantly decreased (all p < 0.001).	Fewer movement artifacts (0% vs 2.8%); possible reduction in FDG uptake in muscles
Tekinhatun et al.^48^	Music listening via speaker system in a specially designed waiting room	Ambient relaxing guitar/piano/flute music	HADS-A, STAI-State, heart rate, beta-blocker use, radiation dose all significantly decreased (p < 0.001).	Imaging rated excellent: 65.7% (DWR) vs 37% (SWR), p < 0.001.
Ma et al.^49^	Converted MRI scanner noise into music using musical gradient sequences	Yo–Yo Ma's Bach Suite No. 1; MRI scanner-generated music	Comfort score for MRF-Music = 7.2 vs 4.5 (EPI) and 4.9 (TSE); p < 0.001.	Improved comfort; high quantitative accuracy; minor efficiency loss
Wen et al.^50^	Music listening delivered via headphones during uptake	Not explicitly stated; likely meditative, relaxing music	Reported improvements in HRV and anxiety based on intervention modelling	Suggested decrease in physiological stress may reduce false positives

BP, blood pressure; DWR, designed waiting room; EPI, echo planar imaging; FDG, fluorodeoxyglucose; HADS-A, Hospital Anxiety and Depression Scale–Anxiety subscale; HRV, heart rate variability; MRF, magnetic resonance fingerprinting; STAI-S, State-Trait Anxiety Inventory–State subscale; STAI-State, State score of the State-Trait Anxiety Inventory; SWR, standard waiting room; TSE, turbo spin echo.

Standardised, methodologically-selected music minimises variability arising from personal preference and avoids triggering painful music-evoked autobiographical memories.^62^, ^63^, ^64^ While standardised music medicine interventions may overcome these limitations and show promising results, many music medicine studies remain limited by subjective or genre-based music selection approaches that lack scientific rigour and reproducibility.

#### Holistic outcomes: quality of life, functional recovery, and emotional resilience

Music-based interventions have demonstrated promising effects on holistic outcomes in cancer care, including quality of life, functional status, and emotional resilience. However, most studies have centred on music therapy rather than music medicine.^65^, ^66^, ^67^ Although evidence indicates music medicine provides immediate symptom relief, its long-term impact remains underexplored, with few studies examining maintenance of benefit and follow-up beyond the acute phase.

Existing music medicine studies are hindered by significant methodological limitations, including ambiguous intervention protocols, heterogeneity in design, and short follow-up periods. Ratcliff et al. (2014) reported low engagement with a self-administered, “as needed” listening regimen, with patients using the provided 30-min music CDs for an average of only 9.9 4-min music recordings over the course of a month.^68^ The lack of prescribed dosing, delayed initiation (28 days after transplant), and absence of adherence monitoring likely missed the acute symptom window, led to lower motivation for participation given potentially reduced baseline patient symptomatology, and compromised mood entrainment and therapeutic impact. Although high attrition is an expected barrier in terminal populations, patients in recovery or early cancer stages represent viable candidates for longitudinal music medicine research if musical considerations, timing, and dosing are appropriately tailored. Future studies should closely tailor intervention design and delivery to best suit the specific needs of the pre-operative and post-operative stages and implement appropriately structured sessions.

## Molecular and neurophysiological underpinnings

Chemotherapy-induced cognitive impairment (CICI), or so-called chemo-brain, affects memory, attention, executive function, and processing speed, with symptoms that can persist for decades post-treatment.^69^ Mechanistically, CICI derives from oxidative stress, neuroinflammation, and disruption of neurogenesis and synaptic plasticity induced by common chemotherapeutic agents. Agents such as cisplatin and doxorubicin generate reactive oxygen species and trigger microglial activation and pro-inflammatory cytokine release (TNF, IL-1β, and IL-6), disrupting hippocampal and prefrontal circuits that are essential for cognition, emotional regulation, and executive function.^70^ Elevated cortisol, neurofilament light chain, and reduced brain derived neurotrophic factor impart chronic neurotoxic stress and impaired neuroplasticity that represent characteristic contributors to CICI.^71^

Within this context, music provides a plausible but still emerging mechanistic framework for investigating whether music-based interventions may counteract these effects through immune modulation and promoting neurogenesis. Accordingly, these proposed neurophysiological pathways should be interpreted as hypothesis-generating frameworks intended to guide future research, rather than as conclusively established mechanisms of cognitive recovery. Conrad et al. (2007) demonstrated that music exposure reduced IL-6 by 83% and epinephrine by 55%, while increasing growth hormone and IL-4 availability, which help promote repair, neuroregeneration, hippocampal neurogenesis, and anti-inflammatory microglial phenotypes.^72^ These metabolic shifts move physiology from a catabolic toward an anabolic, neurorestorative state. Sleep disruption exacerbates CICI by elevating IL-6 and TNF; however, “sleep-inducing” music (0.01–2 Hz; 45–50 dB) has been shown to increase slow-wave sleep by up to 46.2%,^73^^,^^74^ which could increase memory consolidation via hippocampal-cortical communication, facilitate glymphatic clearance of toxins such as amyloid b, and downregulate stress-associated endocrine activity—functions critical for cognition.^75^

At the neural circuit level, music alters brain electrical activity through tempo-induced entrainment. Slow-tempo music promotes theta and alpha rhythms—frequencies tied to relaxation, memory access, and internal focus—in the frontal and parietal cortices, boosting phase-locking value, and restoring default mode network (DMN) stability—often dysregulated in rumination and CICI.^76^^,^^77^ Fast-tempo music enhances beta and gamma activity, enhancing attention, executive functioning, and cognitive control; therefore, this high-frequency activation may be especially useful in counteracting the sluggish cognitive thought and motivational depletion commonly reported in CICI and CRF.^78^^,^^79^ Importantly, these oscillatory effects correspond to neurotransmitter activity and interregional synchronisation, which may alleviate chemotherapy-induced network deterioration. Furthermore, music listening can upregulate gene expression related to cognitive functions and neuroplasticity by upregulating miRNAs crucial for activating neurons, modulating neuronal plasticity, and protecting dopaminergic neurons.^80^ While preliminary, these findings suggest that music may engage molecular and network–level pathways relevant to CICI and CRF, although definitive causal links between music exposure, neuroplastic gene regulation, and cognitive recovery in cancer survivors remain to be established.

Although these pathways are extensively discussed in neurodegenerative diseases like Alzheimer's, they remain underexplored in CICI. Nonetheless, given overlapping mechanisms between these conditions (neuroinflammation, impaired plasticity, hippocampal dysfunction) and the encouraging literature to date, music medicine warrants systematic investigation in cancer survivorship to determine whether these proposed mechanisms translate into measurable cognitive benefit.^81^

### Endogenous opioid system activation and dopaminergic reward system

Music engages core neurochemical systems, particularly the dopaminergic and endogenous opioid system, that can modulate cognitive, emotional, and pain-related processes frequently disrupted in cancer patients. Pleasurable or emotionally salient music activates the mesolimbic dopamine system, eliciting dopamine release in the nucleus accumbens, ventral tegmental area, ventral pallidum, caudate, and orbitofrontal cortex.^81^ This music-induced modulation may support executive function, motivated attention, working memory, and cognitive flexibility—domains often impaired in patients experiencing rumination and CICI. While confirmation is needed, these effects may also be relevant to counteracting amotivation and anhedonia, related in part, to facilitating synaptic plasticity and strengthening neuronal circuits governing goal-directed behaviour.^82^

Theoretical models such as the neural resonance theory and the predictive coding of music framework provide mechanistic explanations for music's engagement of the dopaminergic reward system.^83^ Neural resonance theory posits that emotionally rewarding music stabilises neuro-dynamics through entrainment to constant harmonic structures (e.g., simple integer ratios), whereas predictive coding of music conceptualises music listening as an active inferential process whereby the brain predicts and responds to deviations from expected melodic, harmonic, and rhythmic patterns, generating dopaminergic bursts in the caudate (anticipation) and nucleus accumbens (reward experience).^83^^,^^84^ This interplay between predictability and surprise underlies music's capacity to evoke pleasure and reward while restoring dopaminergic tone—processes particularly relevant for cancer patients with treatment-related disruptions in neurotransmitter systems governing memory, mood, and learning.^85^

Concomitantly, music activates the endogenous opioid system, modulating pain and emotional states.^86^ Functional imaging studies show stimulation of the periaqueductal grey and the anterior cingulate cortex, triggering endorphin release that binds opioid receptors to suppress nociceptive transmission.^87^ These mechanisms likely underly the reduced pain, anxiety, and morphine-equivalent opioid effect in cancer cohorts following MBIs.^54^^,^ (Figure). Music thus holds potential as a non-pharmacological alternative strategy for managing chronic and treatment-related pain.

### Hypothalamic-pituitary-adrenal axis modulation and autonomic nervous system

The human stress response is orchestrated by the hypothalamic-pituitary-adrenal (HPA) axis and the autonomic nervous system. Chronic activation of these systems, common in cancer patients facing prolonged stress or inflammation, elevates cortisol levels that, while adaptive acutely, become deleterious when sustained—promoting immune suppression, neuroinflammation, and pro-inflammatory cytokine release (IL-6, TNF). In effect, this hormonal cascade perpetuates inflammatory processes associated with CRF, pain, tumour growth, metastasis, and CICI often experienced by chemotherapy patients.^85^ Persistent cortisol elevation further reduces natural killer (NK) cell activity, weakening immune surveillance against tumour cells.^88^ However, music has been shown to downregulate HPA axis activity, lowering serum cortisol and buffering the physiological effects of stress,^22^^,^^89^ consistent with findings of reduced sedative requirements following music exposure.^22^

Concurrently, some evidence indicates music may modulate the autonomic nervous system by shifting balance from the sympathetic (fight-or-flight) branch towards parasympathetic (rest-and-digest) state via vagal activation.^90^ This shift towards the parasympathetic state is associated with the release of neurotransmitters like acetylcholine, which can inhibit pro-inflammatory cytokine production (IL-6, TNF) through the cholinergic anti-inflammatory pathway. By reducing sympathetic overactivation, characterised by increased heart rate, blood pressure, and catecholamines, music may help attenuate stress- and inflammation-related symptom burden.^91^ Although these autonomic and inflammatory pathways are biologically relevant to processes implicated in tumour progression, angiogenesis, and metastasis, their direct modulation by music medicine in cancer patients remains an emerging area requiring further investigation.

Mechanistic understanding of music's physiological influence on neural circuitry and the nervous system is critical towards the aim of elevating music from a poorly understood supportive care tool to a more precision-based biologically grounded therapeutic strategy, with progress toward this goal evidenced by recent work reporting effects of music upon inflammation, stress response, and autonomic processes (Table 4). Crucially, continued application of a rigorous, scientifically oriented approach will help position music medicine as a cost-effective and non-pharmacological alternative or adjunct to more traditional methods of symptom management.

**Table 4:** Mapping cancer-related symptoms to underlying biological mechanisms, biomarkers, and music-induced physiological pathways.

	Biological mechanism	Biomarker/Mediator	Music's therapeutic effects
Pain	Nociceptive signalling and inflammation	IL-6, TNF, Substance P, β-Endorphin, CRP, NGF, PGE2	Activates endogenous opioids, reduces IL-6 and TNF, increases β-Endorphin
Cancer-related fatigue	Systemic inflammation and HPA axis dysregulation	IL-6, TNF, IL-1β, Cortisol, CRP, ATP, BDNF	Reduces pro-inflammatory cytokines and cortisol, increases BDNF
Chemotherapy-induced Nausea and Vomiting	Emetic reflex activation via gut-brain signalling	5-HT (Serotonin), Substance P, Dopamine, Cortisol, IL-6	Modulates autonomic nervous system to reduce nausea, lowers cortisol and IL-6
Anxiety	HPA axis hyperactivity and neurotransmitter imbalance	Cortisol, CRH, GABA, Serotonin, Norepinephrine, IL-6, TNF	Reduces cortisol, enhances GABA and serotonin, dampens sympathetic tone
Depressive Symptoms and Mood Disturbances	HPA axis dysregulation, inflammation, neuroendocrine dysregulation	BDNF, Serotonin, Dopamine, IL-6, TNF, Cortisol, IL-1β, CRP	Enhances dopamine and BDNF, reduces pro-inflammatory cytokines
Chemotherapy-induced cognitive impairment (CICI)	Neuroinflammation, oxidative stress, and plasticity deficits	BDNF, VEGF, IL-6, TNF, IL-1β, Cortisol, NfL, ROS	Increases BDNF and VEGF, reduces IL-6 and TNF, improves EEG synchrony
Rumination	DMN hyperactivity and poor executive control	IL-6, TNF, Cortisol, Alpha/Theta/Beta rhythms	Restores DMN function, enhances frontal-parietal connectivity, activates motivational thought and reward system processing

ANS, autonomic nervous system; BDNF, brain-derived neurotrophic factor; CRH, corticotropin-releasing hormone; CRP, C-reactive protein; DMN, Default Mode Network; HPA, hypothalamic-pituitary-adrenal; NGF, nerve growth factor; PGE2, prostaglandin E2; ROS, reactive oxygen species; VEGF, vascular endothelial growth factor.

## Animal models

Preclinical studies in animal models reinforce music's therapeutic potential beyond mood relief, demonstrating reproducible biological effects in tumour suppression, immune modulation, and neuroprotection.^92^^,^^93^ Auditory stimulation has been shown to stabilise the HPA axis, lower cortisol levels, and even facilitate drug delivery across the blood–brain barrier, a major challenge in neuro-oncology^94^, ^95^, ^96^ (Table 5). Furthermore, music reverses stress-induced immunosuppression, restoring NK cell activity and enhancing lymphocyte and T-cell activation, all collectively contributing to reduced tumour mass, volume, and metastatic spread.^92^^,^^97^ Beyond cancer physiology, music exposure reduces anxiety- and depression-like behaviours and supports neural health by promoting hippocampal neurogenesis, preserving dendritic spine density, and reducing oxidative stress markers (e.g., MDA, NO) while boosting endogenous antioxidant enzymes.^93^

**Table 5:** Summary of music medicine interventions in modulating cancer-related symptoms in animal models.

Source	Animal	Music intervention	Music selection	Major findings
Ciborowska et al.^95^	Cattle, poultry, pigs, horses, carps, trout	Mozart, Bach, Vivaldi, lullabies, dinner music, Indian instrumental, and rock'n roll; 1–8 h per day, up to 60 days	60–100 dB depending on species; tempo: 60–200 bpm	Classical music reduced stress, stereotypies, and improved welfare in pigs and cows; rock/harsh music often had negative effects (↓ growth, ↑ stress); music improved immune markers (NK cells, IgG, IL-2, IFN-γ, IL-4)
Fu et al.^93^	Female BALB/c mice, 11–12 weeks old	Baroque, Classical, Romantic, and traditional Eastern/Western styles; 1.5 h per day for 23–28 days	Curated playlist; instruments: piano, harp, violin, guqin; 70 dB	Decrease in depression- and anxiety- like behaviours (NO, malondialdehyde), restored synaptic plasticity (SOD, catalase), preserved neuronal integrity (doublecortin, MAP2)
Gao et al.^92^	Male Wistar rats (5–8 weeks old)	Mozart Sonata K448; played once every hour during the daytime for 2 weeks, 1 week post CT-26 cell injection into tibia	∼60 dB	Significantly reduced cancer-related pain, decreased tumour volume, improved weight gain and feed efficiency, downregulated MAPK pathway proteins
Núñez et al.^97^	Male BALB/c mice (7–12 weeks); Male Sprague Dawley rats (2 months) for cancer	Herbert von Karajan Adagio; (e.g., Mahler, Pachelbel, Vivaldi, Brahms, Mozart)	Classical music CD; <40 dB	↑ lymphocytes, T-cell proliferation, NK cell activity, reduced ACTH, and attenuated metastasis progression. music reduced % metastatic area
Semyachkina-Glushkovskaya et al.^96^	Male Wistar rats (∼2 months old) with glioblastoma	Loud rock music (Scorpions, “Still Loving You”); 1 min on/1 min off for 2 h; on days 7 & 14 post-tumour implantation	100 dB	Music opened the blood–brain barrier, increased bevacizumab delivery, reduced tumor volume and proliferation, improved survival
Barcellos et al.^98^	36 adult wild-type outbred zebrafish	Vivaldi, 2 h, two times a day, 15 days	Classical, pieces from Spring, Concert in C major; 65–75 dB	Reduced anxiety-like behaviour, downregulated pro-inflammatory cytokines (IL-1β, IFN-γ), upregulated BDNF in the brain; no change in cortisol

ACTH, adrenocorticotropic hormone; MAP2, microtubule-associated protein 2; MAPK, mitogen-activated protein kinase; NK, Natural Killer; NO, Nitric Oxide; SOD, Superoxide Dismutase.

Collectively, these findings suggest that music may function as a biologically active intervention capable of modulating depressive behaviours, tumour microenvironments, immune responses, and neuroplastic processes. Although animal models may not encompass the ethological relevance that music has for humans, these studies may inform our understanding of the physiological and molecular changes that may translate to human physiology, underscoring the need for more comprehensive, multimodal music medicine studies investigations in tumour-bearing animal models remain needed.

## Music-based interventions in patients—delivery, dosage, and reproducibility, and future directions

In studies addressing the multifactorial symptoms of cancer, MBIs often rely on generic relaxing music with minimal musician input, selecting tracks based on superficial features (e.g., 60–80 bpm, low timbre) rather than empirically grounded musical parameters. Consequently, few interventions employ standardised protocols linking specific acoustic features to defined clinical outcomes. To address this gap, a collaborative team comprised of a Grammy-nominated composer, musicians, scientists, and musicologists developed the 16 Compositional Elements of Relaxation (CER)—a framework empirically identifying musical parameters (e.g., articulation, accentuation, rubato, register) to perceived relaxation across diverse demographic groups (n = 293).^99^ Unlike genre-based classifications, which are inherently subjective and inconsistent, the CER framework enables replicable, clinically defined method for therapeutic music selection. Efforts to systematise or “prescribe” therapeutic music are not entirely new. Rossetti previously argued that prerecorded music used in clinical settings should be selected through a more deliberate, goal-oriented process rather than by convenience or broad genre labels alone, while the Therapeutic Function of Music Plan provided an ante hoc framework for linking treatment goals and theoretical rationale to specific musical elements.^100^^,^^101^ More recent developments similarly reflect continued interest in structured music prescription, including technology-enabled platforms such as MedRhythms and MediMusic, algorithmic and artificial intelligence (AI)-guided playlist generation, and other emerging affective music-generation systems designed around target emotional or physiologic outcomes. Within this broader landscape, the 16 Compositional Elements of Relaxation (CER) framework may be understood as one additional effort to move beyond generic “relaxing music” labels toward more reproducible, feature-based music selection in music medicine.

Despite encouraging outcomes, clinical translation and long-term, holistic outcomes remain limited by heterogeneity in study design, intervention delivery, and outcome measures with many music medicine studies relying on self-report questionnaires alone that do not capture the underlying physiological mechanisms driving intervention efficacy. Further, these methodological issues are compounded by the often minimal involvement of musicians or input of composers,^102^ absence of defined acoustic parameters,^103^ and use of patient-preferred music, which risks triggering adverse emotional or physiological responses (e.g., emotionally charged lyrics, high-frequency instrumentation, or traumatic memories). However, the relative value of patient-preferred music vs prescribed music remains an open question. While patient-preferred music may enhance familiarity, cultural congruity, and emotional engagement, prescribed or protocol-guided music may offer greater standardisation in linking specific acoustic features to defined physiological outcomes. Future comparative studies are necessary to determine whether, and under what conditions, personalisation improves outcomes relative to more standardised approaches. Such unresolved methodological differences limit reproducibility, complicate cross-study comparison, and hinder efforts to determine best practices for music medicine implementation.

It should be noted that many of the proposed neurophysiological mechanisms discussed in this review are extrapolated from broader literature on music's effects on brain entrainment, autonomic regulation, neuroendocrine activity, immune signalling, and physiological stress responses, rather than conclusively validated in cancer-focused music medicine studies. Accordingly, future formal systematic reviews and meta-analytic syntheses will be necessary to assess study quality, bias, clarify effect sizes, and identify which intervention characteristics are most consistently associated with benefit for specific populations of interest. Specifically, larger scale and more definitive studies are needed, especially those incorporating objective, multimodal assessments—stress biomarkers, fMRI scans, proteomics and metabolomics analyses, and real-time EEG scans—offering a more rigorous, reliable means of validating music's mechanistic effects. Integrating CER-guided music with such tools enhances the scientific rigour, reproducibility, and clinical translatability of music medicine research. Supporting this approach, large-scale research (n = 5000) demonstrated that listener response aligns more closely with intrinsic musical features termed as “mellow” and “sophisticated” rather than genre alone.^104^ Methodical, science-based frameworks like CER enable reproducible, safe, and therapeutically consistent music medicine protocols across clinical populations.

Although major institutions, including the Cleveland Clinic Arts and Medicine, Berklee's Music and Health Institute, and the Louis Armstrong Center at Mount Sinai, have advanced clinical music programs, these efforts primarily emphasise music therapy. Even programs leveraging neuroimaging (e.g., Johns Hopkins Music and Medicine Center) often rely on therapy-based models rather than prescribed musical elements. Despite institutional progress, comparatively little emphasis has been placed on music medicine and systematically characterising music itself as a biologically active stimulus using objective, physiologically grounded endpoints.

This limitation is particularly pronounced in oncology, where symptom burden spans whole-body responses reflected in biomarkers, neuroendocrine activity, and brain function. The Music-in-Medicine Initiative at MD Anderson Cancer Center directly addresses this gap as the first large-scale effort to systematise music selection and evaluate music as a quantitatively oriented, biomarker-driven therapeutic modality. Integrating proteomics, metabolomics, serum and plasma biomarkers, real-time EEG, pupillometry, and single-cell RNA sequencing, the program unites musicians, musicologists, neuroscientists, and computational scientists around CER-based music selection and extends evaluation to caregivers and providers.

Although neurophysiological monitoring in music medicine has become increasingly standardised, music selection remains largely unstandardised. Many studies continue to use loosely defined “relaxing” music without precise and reproducible characterisation of compositional elements and acoustic parameters (e.g., timbre, rhythm, articulation) and biological outcomes. The next frontier lies not only in measuring music's effects but in engineering the music itself with scientific and musical precision. Computational music analysis tools (e.g., MIRtoolbox, Librosa,^105^ Essentia) allow extraction of quantifiable musical features that can be empirically linked to real-time outcomes such as cerebral perfusion, parasympathetic activation, gamma-band (cognition) or alpha-band (relaxation) brain stimulation, or cytokine suppression.

**Fig. 1:**
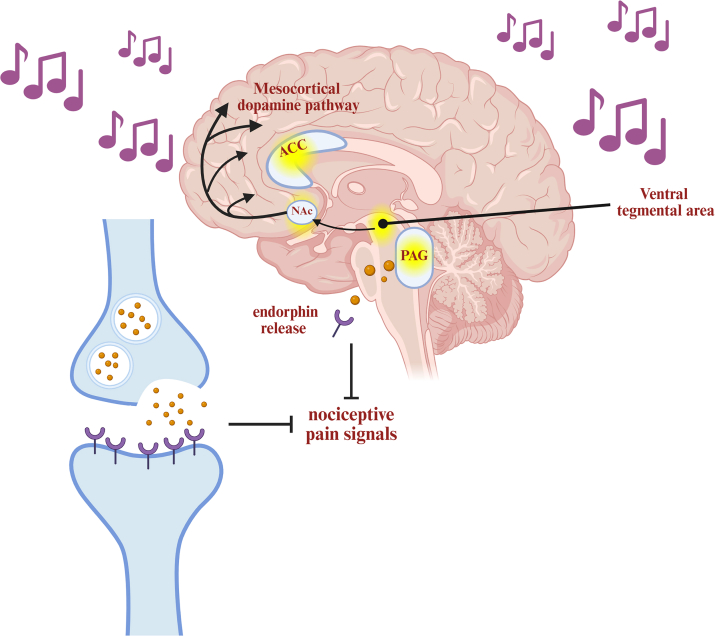
**Neural mechanisms of music-induced analgesia**. Music stimulation engages the mesocorticolimbic reward circuitry, activating the ventral tegmental area (VTA), nucleus accumbens (NAc), anterior cingulate cortex (ACC) and periaqueductal grey (PAG). This dopaminergic pathway promotes endorphin release, suppressing nociceptive pain signalling and resulting in a bidirectional engagement of reward and hedonic pleasure.

AI-integrated, closed-loop systems may eventually tailor music in real-time to sustain therapeutic states and personalise interventions through integration with patient records and treatment plans. Large language models (LLMs) and machine learning approaches can enable *precision music prescriptions* in oncologic care when trained on longitudinal, multimodal data that link patient context—symptoms, treatment phase, environment (OR, ICU, ER, chemotherapy sessions, in–home), preferences, circadian rhythm—and music attributes (acoustic features, familiarity, delivery) to clinically meaningful outcomes, using causal and adaptive learning instead of simple correlations or associations. In practice, LLMs can function as a clinical interface layered over outcome-driven recommender models and safety rules, refined through expert music-therapy supervision and evaluated with the rigour of supportive-care interventions rather than consumer playlist systems (see Fig. 1).

## Conclusions

Music medicine is a promising, non-pharmacological adjunct to conventional cancer care, offering accessible and cost-effective symptom relief for patients experiencing fatigue, pain, and cognitive or affective disturbances—contexts in which active participation in other mind-body therapies may not be feasible. Beyond symptom modulation, growing clinical and preclinical evidence implicates immersive music exposure in the modulation of physiological mechanisms central to cancer progression, including HPA axis activity, immune regulation, and stress response. While promising, confirmation remains needed to determine whether these mechanistic effects translate into durable clinical benefit or meaningful modulation of cancer-relevant biological pathways.

Realising music medicine's full therapeutic potential will require an interdisciplinary, multi-modal paradigm that unites musical expertise, computational modelling, and clinical medicine to truly transform music into a responsive, precise, and clinically actionable intervention. By adhering to a scientifically and methodologically planned approach, music medicine can establish itself as a validated, replicable tool within the standard of care in oncology and beyond.

## Contributors

AH: study design and investigation, methodology, literature search and data curation, writing—original draft, review and editing, visualisation and figures.

MR: conceptualisation, study design and investigation, methodology, writing—review and editing, supervision, project administration.

KN, WJR, FL: writing—review and editing.

All authors have read and approved the final version of the manuscript.

## Declaration of interests

No conflict of interest to declare.

## Appendix A Supplementary data

Music in Medicine Concerts and Data Recording

Dr. Rui Anxiolytic Music Prescription
